# Enhancing Inflammatory Bowel Disease (IBD) competency in gastroenterology fellowship through traditional, digital, and experiential learning pathways

**DOI:** 10.1093/ibd/izag043

**Published:** 2026-03-22

**Authors:** Aimen Farooq, Mariam Naveed, Reezwana Chowdhury, Jennifer Seminerio, Joseph Sleiman

**Affiliations:** Department of Gastroenterology and Hepatology, AdventHealth Orlando, Orlando, FL, United States; Department of Gastroenterology and Hepatology, AdventHealth Orlando, Orlando, FL, United States; Department of Gastroenterology and Hepatology, Johns Hopkins Medicine, Baltimore, MD, United States; Department of Gastroenterology and Hepatology, AdventHealth Orlando, Orlando, FL, United States; Digestive Disease Institute, Cleveland Clinic, Cleveland, OH, United States

**Keywords:** education, inflammatory bowel disease, fellowship

## Abstract

The rapidly evolving field of Inflammatory Bowel Disease (IBD) places growing educational demands on gastroenterology trainees, many of whom report gaps in confidence and preparedness in managing IBD across diverse clinical settings. This article provides a practical, inclusive framework to support gastroenterology fellows in achieving core competency in IBD care while outlining scalable pathways for those interested in developing specialized expertise. Traditional resources such as major conferences, society guidelines, consensus statements, and peer-reviewed journals form the foundation of evidence-based training. Equally vital, however, are opportunities to refine cross-disciplinary skills through close collaboration with radiology, pathology, and endoscopy teams. Visiting observerships at leading IBD centers can be pivotal for immersive, hands-on exposure to complex case management and multidisciplinary care models. The article highlights a growing array of digital and longitudinal learning platforms including webinars, educational websites, podcasts, and programs such as virtual grand rounds, IBD Live, IBD-EII, and IBD REACH that offer flexible, accessible, and globally connected learning experiences. Additionally, it underscores the importance of mentorship, leadership development, and institutional support from program directors in fostering individualized IBD training tracks. By integrating traditional educational foundations with innovative, accessible non-traditional resources and platforms, this guide positions IBD training within a framework of inclusivity, equity, and lifelong learning.

## Graphical Abstract

**Graphical Abstract:**
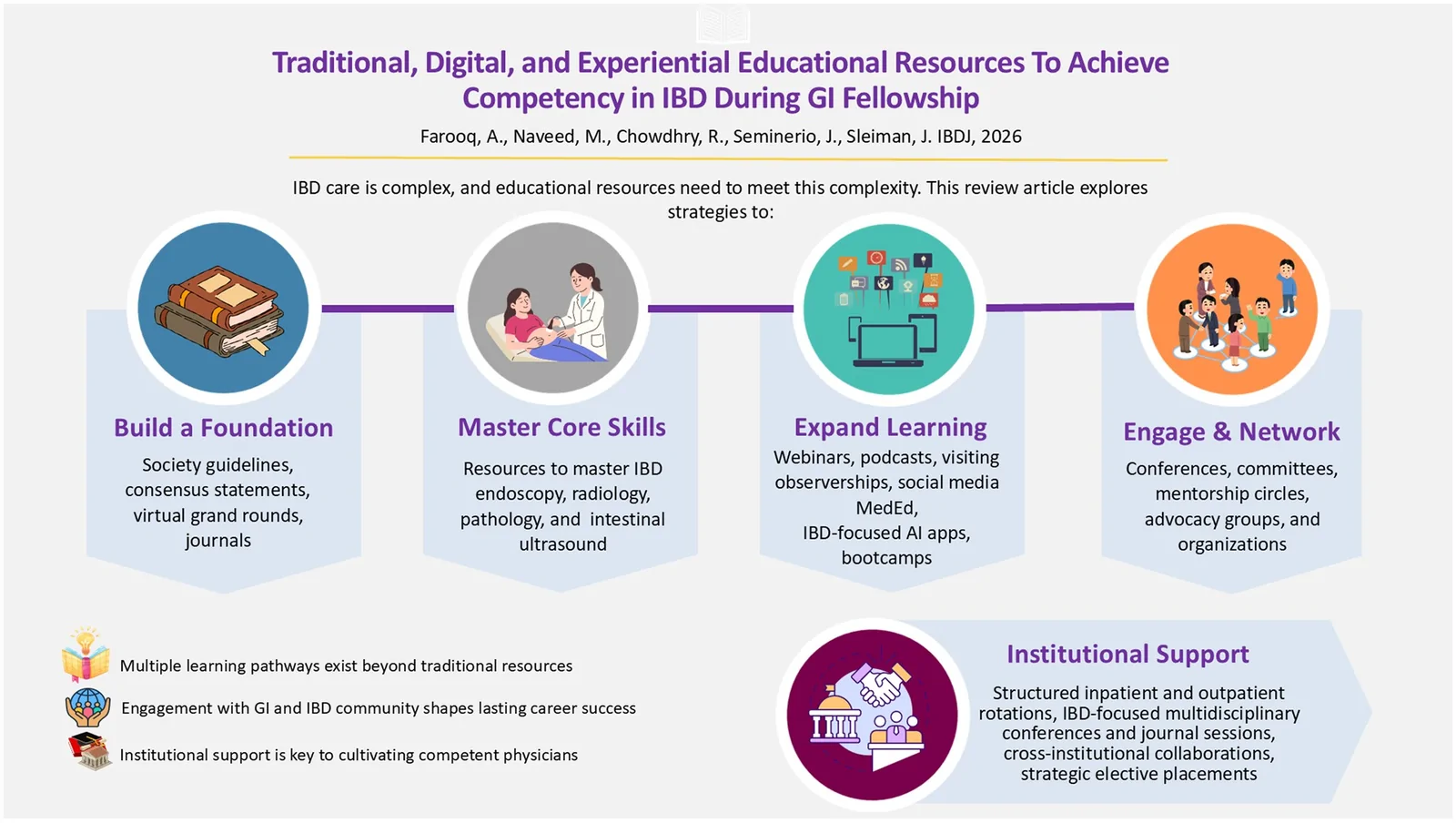

## Introduction

The management of inflammatory bowel disease (IBD) involves the complex knowledge of rapidly expanding therapeutic options and individualizing care regarding extraintestinal manifestations, surgical approaches, mental health disorders, among other multi-disciplinary aspects of care. In response, formal IBD curricula have been developed for gastroenterology (GI) trainees, but more than one-third of trainees remain uncomfortable with their level of IBD training.^1^ Structured clinical sessions can improve knowledge, but fellows still emphasize the need for broader and more clinical experience in IBD management.^2^^,^^3^ Therefore, some trainees pursue a dedicated year of specialized IBD training that incorporates exposure to complex and refractory disease, advanced therapeutics and academic scholarship across medical, procedural and surgical domains. Prior literature has highlighted the value of such fellowships in developing advanced clinical expertise, fostering research productivity, and preparing future leaders in IBD care and research.^4–7^ However, access to advanced IBD training remains constrained by a limited number of fellowship positions, the requirement for an additional year of training, variable funding mechanisms, and application pathways that frequently exist outside traditional accreditation structures. Consequently, many gastroenterology fellows, particularly early in training, seek structured guidance to build IBD knowledge and skills regardless of whether they ultimately pursue additional training. This article is intentionally designed to serve as a foundational framework for IBD education during gastroenterology fellowship. While it may be particularly useful for trainees who do not plan to pursue advanced IBD fellowship but anticipate caring for patients with IBD, the framework is also relevant for early fellows who ultimately elect to pursue subspecialty IBD training. By engaging with publicly available educational tools, mentorship networks, and experiential learning opportunities, this guide aims to equip trainees with the knowledge and skills needed to care for patients with IBD in any practice setting, while providing programs without established IBD centers with a practical, scalable reference to promote competency, confidence, and high-quality, evidence-based IBD care. Although primarily intended for a U.S. audience, the insights and resources presented in this article are highly relevant to global readers.

## IBD-specific entrustable professional activities

Entrustable professional activities (EPA) represent defined tasks and responsibilities a practicing subspecialist should be able to perform independently in their field. The American College of Gastroenterology (ACG) and Crohn’s & Colitis Foundation (CCF) jointly developed and validated a set of EPAs for advanced IBD training (Table 1) that outline the knowledge, skills, and attitudes fellows should achieve by the end of training.^8^

**Table 1:** Entrustable professional activities (EPA) for advanced IBD training developed by ACG and Crohn’s and Colitis Foundation Task Force.

Entrustable professional activities (EPAs) for inflammatory bowel disease training
EPA 1: Classify inflammatory bowel disease phenotype, disease activity, and extraintestinal manifestations
EPA 2: Utilize advanced diagnostic and therapeutic endoscopic and radiographic techniques in the management of inflammatory bowel disease
EPA 3: Prescribe and manage evidence-based inflammatory bowel disease therapies utilizing clinical pharmacologic principles in clinical practice
EPA 4: Manage adverse events and complications of inflammatory bowel disease and inflammatory bowel disease therapies
EPA 5: Manage hospitalized patients with inflammatory bowel disease
EPA 6: Manage pre- and postoperative care for inflammatory bowel disease patients
EPA 7: Manage inflammatory bowel disease in special populations
EPA 8: Recognize the importance of psycho-behavioral health in inflammatory bowel disease and implement psychosocial support strategies
EPA 9: Evaluate and manage nutritional health status in inflammatory bowel disease patients
EPA 10: Apply preventive health strategies for inflammatory bowel disease patients in accordance with guidelines

Adapted from Cohen et al.^8^

Early guidance for incoming fellows should focus on defining foundational competency versus advanced specialization. All gastroenterology fellows, regardless of ultimate career focus, should graduate with the core skills aligned with proposed IBD EPAs for categorical gastroenterology fellows (Table 2) that represent a minimum competency threshold for independent practice.^9^ Fellows interested in an IBD-focused career should aim to master the basic and advanced competencies to demonstrate readiness for independent, expert-level practice.^8^^,^^9^ For trainees at programs with established IBD expertise, structured exposure through continuity clinics, multidisciplinary conferences, and elective time can help determine whether deeper specialization or an advanced IBD fellowship year is warranted. In contrast, fellows at programs with limited subspecialty expertise may benefit from early engagement with national curricula, digital case-based learning platforms, and visiting observerships to supplement local training.

**Table 2:** Proposed IBD entrustable professional activities (EPA) for general gastroenterology fellows.

Proposed IBD entrustable professional activities (EPAs) for general gastroenterology fellows
1: Diagnose IBD and identify disease type, extent, and behavior (Crohn’s disease)
2: Recommend and interpret results of appropriate diagnostic testing in IBD
3: Differentiate mild IBD from moderate-severe IBD and recommend appropriate therapy
4: Understand when and how to refer to an IBD specialist for moderate-severe IBD if a local expert is not available
5: Differentiate between activity and severity in IBD
6: Recommend appropriate preventative health care for IBD patients
7: Recognize the importance of the interdisciplinary team in IBD care including physicians, advanced practice providers, surgeons, nutritionists, mental health providers if and when available
8: Manage IBD patients including recognizing when a patient should be transferred to an IBD center for urgent care

Adapted from Malter et al.^9^

## Traditional educational sources

GI professionals often rely on society guidelines, consensus statements, recommendations, and clinical practice updates as foundational resources for IBD management. These societies and organizations include the American Gastroenterological Association (AGA),^10^ the ACG,^11^ International Organization for the Study of IBD (IOIBD), and the international collaboration through the European Crohn’s and Colitis Organization (ECCO).^12^ Topics related to endoscopic techniques are often addressed by the American Society for Gastrointestinal Endoscopy (ASGE)^13^ and the European Society of Gastrointestinal Endoscopy (ESGE).^14^ Pediatric guidelines are covered by the North American Society for Pediatric Gastroenterology, Hepatology and Nutrition (NASPGHAN), the European Society for Pediatric Gastroenterology, Hepatology and Nutrition (ESPGHAN), and the American Academy of Pediatrics (AAP).^15–17^ Readers should note the year of publication for each guideline and be aware that some are living documents that undergo regular updates.^18^

Geography-specific guidance has also been published by regional societies, including Asia-Pacific Working Group on IBD (under the Asia-Pacific Association of Gastroenterology and the Asian Organization for Crohn’s and Colitis), the Asian Pan-Pacific Society for Pediatric Gastroenterology, Hepatology, and Nutrition (APPSPGHAN), Pan American Crohn’s and Colitis Organization (PANCCO), the Emirates Gastroenterology and Hepatology Society, and the Italian Group for the Study of Inflammatory Bowel Disease. Topic-specific expert consensus statements have emerged from groups such as the Strategic Alliance for Intercultural Advocacy in GI (SAIA), which addresses cultural competency in South Asian IBD patients^19^ and the Pregnancy in IBD and Neonatal Outcomes (PIANO) study group, which guides IBD management during pregnancy.^20^

While there are many general GI journals, fewer are dedicated exclusively to IBD, including Inflammatory Bowel Diseases (IBD), Journal of Crohn’s and Colitis (JCC), and Crohn’s and Colitis 360 (CC 360). Engaging with these journals not only deepens clinical and research knowledge but also creates opportunities for professional growth through editorial fellowships, peer reviews, and editorial experiences.

In addition, PubMed offers a robust, powerful alert system that helps users stay updated on the latest research in IBD, customized to their specific interests. Alerts can be created by selecting appropriate Medical Subject Headings (MeSH) terms and applying relevant filters to ensure targeted results.

## Endoscopy, pathology, and imaging skills

IBD providers are expected to develop distinct procedural and interpretive skills. Specialized IBD endoscopic techniques include screening for dysplasia via high-definition white light or chromoendoscopy, stricture dilation, and pouch evaluation. IBD providers regularly assess histopathological features to rule out IBD, including architectural distortion, patterns of inflammation, and granulomas. Understanding different imaging modalities and their use in assessing fistula or abscess formation, transmural thickening, strictures, and surgical anatomy is also essential for IBD care. Cross-sectional imaging may also be used to detect relevant extraintestinal features, such as spondyloarthropathies and muscle loss.

If gaps in local training curricula exist, Table 3 summarizes available online resources to enhance endoscopy, imaging, and pathology skills. Trainees should also proactively identify shadowing experiences, either regionally or by applying to visiting observerships.

**Table 3:** Web-based educational resources for endoscopy, radiology, and pathology.

Resource	Description	Website/URL
**Endoscopy**		
**EndoCollab**	Online community of endoscopists with skill-enhancement and educational resources.	Home—EndoCollab https://endocollab.com
**EndoscopyNOW**	Mobile platform for up-to-date, expert-driven procedural insights.	EndoscopyNow https://endoscopynow.com
**Endoscopy in IBD** ** (Endoscopy on air)**	Global 4-hour live streaming event.	Endoscopy On Air—Global Endoscopy Virtual Events https://endoscopyonair.com
**IBDscope.com**	Dedicated IBD endoscopy resource.	IBDscope—Medical news on IBD
**YouTube channels**	Educational content from global experts and societies.	YouTube https://www.youtube.com
**Radiology**		
**RSNA radiology case collection**	Curated radiology case collection by Radiologist Society of North America.	Home | RSNA Case Collection https://cases.rsna.org
**ECCO/ESGAR e-workshops**	Online workshops for GI radiology and IBD imaging.	ECCO e-Learning Platform https://www.ecco-ibd.eu/education/e-learning
**IBUS**	Real-world global IUS cases with Q&A sessions and expert panel.	Webinars—IBUS—International Bowel Ultrasound Group
**iUSCAN**	Real-world IUS cases with Q&A sessions from the United States and Canada.	Intestinal Ultrasound Learning Center | Articles, Webinars, Training
**Pathology**		
**PathologyOutlines.com**	Free, no-registration online pathology reference website.	Pathology Outlines—PathologyOutlines.com https://www.pathologyoutlines.com
**WebPath**	Online pathology teaching resource (University of Utah).	Pathology images and text for medical education—WebPath https://webpath.med.utah.edu
**Virtual Microscopy Database**	A centralized database of digital pathology slides maintained by a group of histology educators.	Virtual Microscopy Database https://www.virtualmicroscopydatabase.org
**IBDebrief**	Grant-funded multidisciplinary educational program to enhance IBD knowledge among community. gastroenterologists hosted by Icahn School of Medicine.	IBDebrief Cases—YouTube https://www.youtube.com/playlist?list=PLqLDR0CTP9_rBwBUo3ej_RmYg9V7LPBkC

More recently, intestinal ultrasound (IUS) has emerged globally as a validated, noninvasive, and cost-effective imaging modality for IBD.^21^ Learning IUS equips clinicians with an in-clinic tool that aids in comprehensive IBD monitoring, aligning with modern treat-to-target strategies. Fellows can develop proficiency in IUS through membership in the International Bowel Ultrasound Group (IBUS), which provides a 3-module pathway to certification and eligibility to apply for ECCO-IBUS research grants and collaborative scientific projects.^22^ For North American trainees, the Intestinal Ultrasound Group of the United States and Canada (iUSCAN) provides similar training opportuinities.^23^

## Educational platforms and digital learning tools

While UpToDate (Wolters Kluwer)^24^ served as the primary source of evidence-based information for healthcare providers over the past two decades, newer IBD-focused platforms are emerging. IBD-EII is a free website that summarizes IBD clinical trials in a timeline fashion, shares helpful IBD “cheat sheets,” and serves as a repository for global IBD guidelines. It is suitable to support day-to-day clinical decision-making and self-paced learning.^25^

Artificial intelligence-driven platforms (eg, ChatGPT, Gemini, Claude) are useful tools for quick answers, yet some versions can “hallucinate” inaccurate content or references, so caution must be taken. Among medically oriented engines, OpenEvidence.com is the leading AI-based platform to meet such needs.^26^ Other platforms include Doximity GPT (DoxGPT), and ClinicalKey AI.

Social media platforms, particularly X, Bluesky, and LinkedIn, are powerful educational ecosystems that enhance clinical acumen, help trainees stay abreast with emerging updates, and allow active participation in the IBD community. With spaces such as #MondayNightIBD, #EvidenceBasedGI, #CGH4ALL, trainees can access expert-led discussions, live journal clubs, infographics, continuous medical education (CME) credit opportunities, and real-time updates from major conferences and journals.^27–29^ These platforms, as well as reliable experts in IBD who have a professional SoMe presence, help foster a community of learning, mentorship, and collaboration among providers, patients, and advocacy groups.

Many societies, institutions, pharmaceutical industries, and CME companies offer trainees the ability to watch webinars and multidisciplinary team (MDT) meetings, involving gastroenterologists, colorectal surgeons, radiologists, pathologists, and other IBD-focused specialists. Fellows get firsthand exposure to complex case discussions and collaborative decision-making processes that enrich their understanding of real-world IBD management strategies. A non-exhaustive list is included in Table 4.

**Table 4:** Web-based educational platforms and digital learning tools.

Resource	Description	Website/URL
**Virtual Grand Rounds by AGA University, ACG, and other societies**	On-demand webinars for trainees. Some may require membership registration.^10^^,^^11^	ACG Virtual Grand Rounds | ACG AGA University | Your hub for GI education
**IBD 101 primer course and Mentorship Circle for GI fellows**	A joint New York University Langone and ACG one-day educational course designed for first-year GI fellows, which requires invitation by the program directors. Fellows gain access to ongoing email-based mentorship circle in which participants can pose real-time clinical questions based on patient cases and receive tailored, case-specific feedback from experienced mentors. It is an excellent resource for those seeking real-world mentorship outside of formal training settings.^30^	IBD 101: A Primer for First-Year Gastrointestinal (GI) Fellows | ACG
**IBD LIVE**	A weekly one-hour interactive webcast that features difficult cases from expert IBD centers around the world. Fellows can present cases, gain management insights, and earn CME credits all at once.^31^	IBD Live—ConsultQD Live | Physician to physician video platform
**Virtual Global IBD Visiting Professorship**	A Cleveland Clinic quarterly event that presents lectures and case discussions led by global IBD experts and focuses on the latest research and clinical practices.	Global IBD Visiting Professorship—ConsultQD Live | Physician to physician video platform
**Mentoring in IBD**	A Canadian platform that has been providing educational content for more than 25 years, including a newsletter, IBD Dialogue.	Mentoring In IBD
**IBDIQ.com**	On-demand education platform with curated IBD specialists discussing fundamental and advanced topics in IBD, supported by an educational fund from Takeda.	IBDIQ: IBD Education for Healthcare Professionals
**Gutflix Platform**	Recorded UEG Week lectures, webinars, podcasts, guidelines, clinical courses, and other series to support IBD and gastroenterology learning globally, hosted by United European Gastroenterology.	Gutflix—UEG’s interactive recommendation platform for gastroenterology content.
**IBD Update**	Summaries of IBD research after national and international conferences.	Login | IBD Update
**Cornerstones Health**	Non-profit organization that enhances and transforms communication among healthcare providers, patients, and industry partners through creative, comprehensive, and expert-based educational programs. Summaries of IBD research after national and international conferences.	Home—Cornerstones Health
**YoungIBD.org**	The Young Global IBD Group site established in 2024 for global education and insights in IBD management.^32^	YOUNG GLOBAL IBD GROUP—Young IBD

Several evidence-based IBD focused podcasts offer high-yield discussions led by experts in the field, including but not limited to: Atomic IBD; More than Guts, produced by Bristol Myers Squibb, which features multidisciplinary perspectives from clinicians and researchers on emerging therapies and holistic care; About IBD, which bridges patient and provider viewpoints to highlight real-world management strategies; and Gut Feelings, hosted by Crohn’s and Colitis Foundation dietitians, which explores diet-based strategies grounded in current research.

CME-based platforms that require sign-up and can be tailored for GI or IBD-focused topics include medscape.org, practicingclinician.com, CME outfitters, MyCME, GastroCE, consultant360.com, HealioGI, clinicaloptions.com, Gastroenterology & Endoscopy news (The Regueiro Report). Finally, subscribing to newsletter for organizations, journals, and resources above can help you stay up to date and receive the information straight to your inbox.

Given the rapid evolution of digital tools, trainees and mentors should prioritize resources that demonstrate clear Editorial Oversight (peer-review process or expert board); Society Endorsement (affiliation with organizations like AGA, ACG, ECCO); Recency of Updates; and an Evidence-Base (direct citation of primary literature or clinical guidelines).

## Longitudinal educational programs

Multiple societies offer spaces for continuous education with a focus on IBD. Examples include:

- *Visiting IBD observerships* are offered by CCF whereby second- and third-year GI fellows who lack local IBD training can visit IBD centers of excellence for a duration of one month, with most expenses paid by the foundation.^33^
- *REACH-IBD*, the CCF’s early-career program, is a valuable resource for trainees that offers targeted educational and career development webinars, journal clubs, advocacy, and mentorship opportunities with leaders in the field.^34^
- *IBD clinical trial bootcamp* is offered by AGA for trainees and early career faculty who are interested in harnessing skills for launching IBD clinical trials in their future career, with a particular focus on equity and diversity.^35^
- *Y-ECCO membership* (under age 35) gives trainees free access to JCC and ECCO online learning platforms and eligibility to participate in ECCO guidelines, fellowships, grants, and travel awards.

## Key conferences in IBD

Attending national (Table 5) and international (Table 6) conferences is another component of professional growth for fellows and early-career gastroenterologists pursuing a career in IBD. These gatherings create unique opportunities for hands-on learning, mentorship, and networking with leaders in the field. Whether through poster presentations, case-based discussions, or interactive workshops, conferences provide a platform to deepen clinical knowledge, refine academic interests, and build lasting professional relationships that can shape the trajectory of an IBD-focused career.

**Table 5:** National conferences for IBD education.

Conference name	Typical dates	Common locations	IBD-focused educational opportunities	Trainee opportunities (IBD-specific)	Format
**Digestive Disease Week (DDW)**	May (eg, May 2-5, 2026)	Rotates; 2026 in Chicago, IL	IBD-focused symposia, expert panels, and poster sessions; significant IBD research presence	Present IBD abstracts, network with IBD researchers, attend IBD trainee sessions	In-person
**Advances in IBD (AIBD)**	December (eg, December 7-9, 2026)	Orlando, FL	In-depth clinical case discussions, guidelines updates, and real-world IBD management strategies	Fellows Ambassador Program, IBD trainee meetups, IBD poster sessions, pre-conference workshops	In-person
**Crohn’s & Colitis Congress (CCC)**	January (eg, January 22-24, 2026)	Rotates; 2026 in Las Vegas, NV	Multidisciplinary IBD sessions, cutting-edge therapy updates, and translational science	Podium presentations, junior investigator reception, abstract opportunities	In-person
**GUILD Conference**	February (eg, February 15-18, 2026)	San Juan, Puerto Rico	Focused sessions on IBD treatment challenges, pediatric IBD, and research advancements	Fellow Scholars Program includes IBD-focused mentoring and networking	In-person
**ACG Annual Scientific Meeting**	October (eg, October 9-14, 2026)	Rotates; 2026 in Nashville, TN	Dedicated IBD paper and poster sessions, clinical symposiums, guideline updates	IBD abstract presentations, trainee receptions, Women in IBD and career panels	In-person

**Table 6:** Examples of international conferences for IBD education and research.

Conference name	Typical dates	Location	Focus areas	Travel grants	Why attend
**ECCO Congress (European Crohn’s and Colitis Organisation)**	February	Rotates across Europe (eg, Stockholm 2026)	Comprehensive IBD coverage: clinical practice, research, imaging, surgery, pediatrics	Yes—fellowships, grants, and travel Awards for early-career professionals under forty	Premier IBD-focused conference with extensive networking, educational sessions, and research opportunities
**UEG Week (United European Gastroenterology)**	October	Rotates across Europe (eg, Barcelona, Spain 2026)	Broad GI topics with significant IBD content; includes basic science, clinical research, and translational medicine	Yes—510 travel grants up to €750 for young GI professionals under forty	Offers a wide-ranging GI perspective with opportunities to engage in IBD-specific sessions and collaborations
**World Congress of Gastroenterology (WCOG)**	Every two years	Rotates globally (eg, New Delhi, India 2026)	Global GI topics with sessions on IBD; emphasis on education and training	Yes—Travel grants available to support attendance from various regions	Ideal for fellows seeking a global perspective on GI diseases, including IBD, opportunities to connect with international experts
**IOIBD Meetings (International Organization for the Study of Inflammatory Bowel Disease)**	Semi-annual, invitation only.	Rotates globally	Advanced IBD research, clinical guidelines, and collaborative projects	Yes—Travel grants for young scientists from countries with evolving IBD care	High-level IBD research and consensus-building; suitable for fellows interested in academic and research careers

Regional IBD conferences often hosted by academic institutions or local chapters of national organizations (eg, Advances in IBD regionals) offer accessible, high-yield educational opportunities tailored to fellows and early-career providers. Nearly every academic IBD center has launched some form of summit for updates in IBD. Examples include the IBD Horizons, Precision Medicine in IBD Summit, Allegheny Health Network IBD Summit, and the Annual Mount Sinai Nutrition and IBD Education Session, but the list is increasing yearly. These conferences frequently include in-person and virtual options, making them especially practical for fellows with clinical obligations. Some, like the ACG IBD School and IBD UNITE, integrate patient-centered and advocacy-driven content to enhance the understanding of real-world challenges in IBD care. Additional specialty-adjacent conferences such as American Society of Colon and Rectal Surgeons (ASCRS) and American Society of Clinical Oncology (ASCO) may be valuable to gain deeper insight into surgical and oncology care in patients with IBD. Attending a mix of regional and national conferences can help create a well-rounded educational experience while expanding one’s professional network across geographic levels.

## Mentorship and leadership opportunities

Many resources exist to provide GI trainees with IBD mentorship and leadership opportunities. National organizations including ACG, AGA, CCF, ECCO, ASGE offer IBD-focused content, mentorship, and committee involvement. Programs like the IBD 101 Mentorship Circle, Scrubs & Heels, and social media-based medical education (SoMe MedEd) collaborations provide ongoing, peer-supported learning, and career development. Collaborative research projects are available for those interested in research and quality improvement, such as IBD Qorus, via such organizations. While research collaborations often require a local faculty member to serve as principal investigator for IRB approval, trainees are often given ample opportunities to shine and connect through such collaborations.

Fellows should also explore visiting fellowships and observerships offered by individual institutions or foundations, mainly the CCF IBD visiting fellowship, to gain exposure to high-volume IBD centers and academic environments. Early-career networks such as REACH IBD, Milestone IBD, AGA’s Clinical Trialist Bootcamp, YOGI IBD, Youth-ECCO (Y-ECCO), and IOIBD’s trainee programs offer structured mentorship, hands-on training, and leadership-building opportunities. Meanwhile, advocacy-driven groups like CCF, Strategic Alliance for Intercultural Advocacy in GI (SAIA), and Mind Over Microbiome (MOM) empower fellows to engage in public education, equity in care, and patient-centered initiatives.

## Integrating IBD training within general gastroenterology fellowships

Early exposure, structured mentorship, and program flexibility within the core fellowship provide the foundation for advanced skill development. Although individual initiative remains essential, program leadership plays a defining role in shaping this trajectory. Program directors (PDs) are positioned to establish the curricular structure, mentorship, and collaborative opportunities necessary to transform early interest into focused competence. Achieving this goal requires deliberate program design that integrates IBD exposure, promotes cross-institutional collaboration, and ensures equitable access to advanced training pathways for all fellows, regardless of institutional size or resources.

As subspecialization within gastroenterology expands, there is also a growing need to preserve engagement in IBD education among fellows who may not pursue advanced IBD training. While IBD management is increasingly complex and non-algorithmic, disengagement risks graduating physicians who are underprepared to manage a prevalent chronic disease. Program directors and educators should therefore emphasize graduated responsibility and milestone-linked assessments to ensure that all fellows achieve competence in IBD care, while allowing interested trainees to pursue deeper specialization through elective and longitudinal pathways.

Within this framework, general GI fellowships play a vital role in nurturing IBD interest, even without a dedicated IBD year. Programs can build early exposure through structured inpatient consult rotations, continuity clinics that include IBD patients, and participation in multidisciplinary case discussions with surgery, radiology, and nutrition. Didactic sessions and journal clubs focusing on complex IBD topics reinforce core knowledge and clinical confidence. Elective time should be used strategically, allowing motivated fellows to pursue focused IBD rotations, research projects, or attendance at national IBD meetings.

Integrating IBD-related EPAs into the fellowship curriculum helps ensure consistent competency milestones across programs. Programs can objectively assess IBD competency through case-based discussions, milestone-linked EPA evaluations, pre/post knowledge testing for foundational EPAs, and direct observation or simulation-based assessments for advanced procedural EPAs. Program directors can supplement local education through national learning initiatives such as IBD 101, REACH-IBD, and IBD LIVE, which provide structured curricula, mentorship, and peer networks. Participation in these programs, combined with the use of digital CME resources ensures equitable access to high-quality education regardless of institutional size or IBD volume. These principles apply equally to advanced practice providers, who are increasingly integral to IBD care.

For program directors who may not be closely connected to the national IBD community, building partnerships is key. ECCO, CCF, AGA, ACG, and other national organizations offer trainee mentorship programs, PD networks, and IBD-focused committees that welcome collaboration. Encouraging trainees to attend Digestive Disease Week (DDW), Advances in IBD (AIBD), or the Crohn’s & Colitis Congress provides opportunities to meet educators, identify visiting electives, and connect fellows to mentors. PDs can also engage through the ACG Training Directors Committee and the AGA Training Directors Workshop, which frequently address subspecialty exposure and mentorship development.

When programs lack dedicated IBD faculty or high-volume exposure, PDs can bridge gaps through observerships, regional partnerships, or flexible elective design. Facilitating participation in the CCF Visiting IBD Fellow Program or remote mentorship with faculty at academic centers provides valuable hands-on and longitudinal learning. Encouraging scholarly work (ie, case reports, review articles, or collaborative research) further supports professional growth and visibility within the field.

While a dedicated IBD fellowship offers immersive clinical experience, mentorship, and research training, it remains a non-accredited pathway. With deliberate mentorship, structured elective time, and creative engagement in multicenter or registry-based projects, general GI fellows can build fulfilling, IBD-focused careers. Ultimately, the program director’s role is to ensure access to expertise, mentorship, and opportunity, so that every fellow, regardless of institutional resources, develops both competence in general gastroenterology and the foundation for a rewarding career in IBD.

## Limitations

This review has some important limitations. First, the purpose of this manuscript is to provide a pragmatic roadmap to available training opportunities. While the highlighted programs represent widely recognized resources, the selection is based on expert consensus among the authors and established society presence rather than a comparative assessment of educational interventions. Second, access to certain opportunities such as observerships, national conferences, or high-volume IBD centers may be limited by geography, institutional resources, or funding. To address this, we intentionally emphasize scalable and low-cost alternatives, including digital learning platforms, virtual case conferences, and open-access educational content that can supplement local training environments. Finally, digital tools and educational platforms evolve rapidly; therefore, this review should be viewed as a conceptual framework for engaging with IBD education rather than an exhaustive or static list of resources.

## Conclusion

In 2026, GI fellows can pursue an advanced IBD fellowship for further education and leadership opportunities, but non-traditional avenues exist to fulfill such goals. Pursuing an advanced IBD fellowship year should be individualized and guided by career goals, desired clinical complexity, research interests, and access to mentorship rather than viewed as a mandatory credential. Ultimately, the path to becoming a skilled IBD provider lies in being proactive, resourceful, and community minded. The development of future IBD specialists begins with early curiosity, is strengthened by structured mentorship, and is sustained through supportive training environments. As IBD care continues to evolve, collaboration between fellows, mentors, and program directors will remain central to shaping confident, competent, and compassionate clinicians and researchers. Through shared commitment and access to equitable educational opportunities, the next generation of gastroenterologists can continue advancing excellence in IBD care.

## References

[1] Cohen BL , Ha C, Ananthakrishnan AN, Rieder F, Bewtra M. State of adult trainee inflammatory bowel disease education in the United States: a national survey. Inflamm Bowel Dis. 2016;22:1609-1615. 10.1097/MIB.0000000000000766 PMC4911816 27306068

[2] Lee AJ , Kraemer DF, Smotherman C, Eid E. Providing our fellows in training with education on inflammatory bowel disease health maintenance to improve the quality of care in our health care system. Inflamm Bowel Dis. 2016;22:187-193. 10.1097/MIB.0000000000000573 26332311

[3] Wolff MJ , Balzora S, Poles M, et al Objective structured clinical examination as a novel tool in inflammatory bowel disease fellowship education. Inflamm Bowel Dis. 2015;21:759-765. 10.1097/MIB.0000000000000317 25633560

[4] Falloon K , Regueiro M. Who should consider IBD fellowship? Crohns Colitis 360. 2024;6:otae030. 10.1093/crocol/otae030 PMC11247403 39010946

[5] Hirten R , Sands BE, Cohen BL. The whys and hows of fourth-year inflammatory bowel disease fellowships. Dig Dis Sci. 2017;62:1116-1118. 10.1007/s10620-017-4555-5 PMC6658184 28365918

[6] Gallinger Z. Why you should consider an advanced training fellowship in inflammatory bowel disease. ACG Case Rep J. 2017;4:e15. 10.14309/crj.2017.15 PMC5291714 28184372

[7] Cushing KC , Adar T, Ciorba M, Ananthakrishnan AN. Assessment of benefit of advanced inflammatory bowel disease training: challenges and solutions. Crohns Colitis 360. 2020;2:otaa019. 10.1093/crocol/otaa019 PMC8607547 34825180

[8] Cohen BL , Gallinger ZR, Ha C, et al Development of entrustable professional activities for advanced inflammatory bowel disease fellowship training in the United States. Inflamm Bowel Dis. 2020;26:1291-1305. 10.1093/ibd/izaa177 32820340

[9] Malter LB , Israel A, Rubin DT. Proposal to update the curriculum in inflammatory bowel diseases for categorical gastroenterology fellows. Inflamm Bowel Dis. 2019;25:1443-1449. 10.1093/ibd/izz107 31115448

[10] American Gastroenterological Association Clinical Guidelines. https://gastro.org/clinical-guidance/

[11] American College of Gastroenterology Guidelines. Available from: ACG Guidelines | ACG.

[12] European Crohn’s and Colitis Organisation Guidelines. Available from: ECCO Guidelines—ECCO—European Crohn’s and Colitis Organisation.

[13] American Society of Gastrointestinal Endoscopy guidelines. Available from: ASGE | Practice Guidelines - Standards of Practice.

[14] European Society of Gastrointestinal Endoscopy guidelines. Available from: Guidelines | ESGE.

[15] North American Society for Pediatric Gastroenterology, Hepatology and Nutrition (NASPGHAN) clinical guidelines and position statements. Available from: NASPGHAN - Clinical Guidelines & Position Statements.

[16] The European Society for Pediatric Gastroenterology, Hepatology and Nutrition (ESPGHAN) clinical guidelines and position statements. Available from: ESPGHAN Guidelines.

[17] American Academy of Pediatrics guidelines. Available from: Clinical Practice Guidelines | American Academy of Pediatrics.

[18] Singh S , Loftus EV Jr, Limketkai BN, et al; AGA Clinical Guidelines Committee. Electronic address: clinicalpractice@gastro.org AGA living clinical practice guideline on pharmacological management of moderate-to-severe ulcerative colitis. Gastroenterology. 2024;167:1307-1343. 10.1053/j.gastro.2024.10.001 PMC12162142 39572132

[19] Pandit AU , Tomasino KN, Aswani Omprakash T, Epstein DE. Cultural considerations in gastroenterology: barriers to care and a call for humility and action. Transl Gastroenterol Hepatol. 2024;9:74. 10.21037/tgh-24-17 PMC11535788 39503037

[20] Mahadevan U , Seow CH, Barnes EL, et al Global Consensus Group for Pregnancy and IBD. Global consensus statement on the management of pregnancy in inflammatory bowel disease. Inflamm Bowel Dis. 2025;31:2615-2664. 10.1093/ibd/izaf171 40874613

[21] Chavannes M , Dolinger MT, Cohen-Mekelburg S, Abraham B. AGA clinical practice update on the role of intestinal ultrasound in inflammatory bowel disease: commentary. Clin Gastroenterol Hepatol. 2024;22:1790-1795.e1. 10.1016/j.cgh.2024.04.039 39001773

[22] IBUS Accredited Curriculum; Available from: Education—IBUS—International Bowel Ultrasound Group.

[23] Educational Resources on Intestinal Ultrasound ; Available from: Revolutionizing IBD Care with Intestinal Ultrasound | iUSCAN.

[24] UpToDate. Available from: Evidence-based Clinical Solutions for Healthcare | UpToDate | Wolters Kluwer.

[25] IBD-EII. Available from: IBD-EII | This platform is intended to help share evidence-based data regarding inflammatory bowel diseases in a summarized way.

[26] Open Evidence. Available from: OpenEvidence.

[27] MondayNightIBD. Available from: https://x.com/mondaynightibd?s=21&t=EfCxImUHNtDYanLj0e1u0w

[28] ACG Evidence-Based GI. Available from: https://x.com/acg_ebgi? s=21&t=EfCxImUHNtDYanLj0e1u0w

[29] #CGH4ALL. Available from: https://x.com/aga_cgh?s=21&t=EfCxImUHNtDYanLj0e1u0w

[30] Malter L , Hong SJ, Lopatin S, et al Increasing exposure to inflammatory bowel diseases education in gastroenterology fellowship: the pilot IBD 101 experience. Inflamm Bowel Dis. 2025;31:746-750. 10.1093/ibd/izad311 38217539

[31] Regueiro MD , Greer JB, Binion DG, et al IBD LIVE Physician Group. The inflammatory bowel disease live interinstitutional and interdisciplinary videoconference education (IBD LIVE) series. Inflamm Bowel Dis. 2014;20:1687-1695. 10.1097/MIB.000000000000018 Available from: IBD Live - ConsultQD Live | Physician to Physician video platform 25167213

[32] Young Global IBD Group. Available from: YOUNG GLOBAL IBD GROUP - Young IBD.

[33] Visiting IBD Fellow Program by Crohn’s and Colitis Foundation. Available from: Visiting Inflammatory Bowel Disease Observership Program | Crohn’s & Colitis Foundation.

[34] REACH_IBD. Available from: REACH-IBD: Rising Educators Academics and Clinicians Helping IBD | Crohn’s & Colitis Foundation.

[35] GI Clinical Trialists Bootcamp. Available from: GI Clinical Trialists Bootcamp - American Gastroenterological Association.

